# Alterations of the Ca^2+^ signaling pathway in pancreatic beta-cells isolated from db/db mice

**DOI:** 10.1007/s13238-014-0075-7

**Published:** 2014-07-24

**Authors:** Kuo Liang, Wen Du, Jingze Lu, Fei Li, Lu Yang, Yanhong Xue, Bertil Hille, Liangyi Chen

**Affiliations:** 1 Department of General Surgery, XuanWu Hospital, Capital Medical University, Beijing, 100053 China; 2 National Key Laboratory of Biomacromolecules, Institute of Biophysics, Chinese Academy of Science, Beijing, 100101 China; 3 The State Key Laboratory of Biomembrane and Membrane Biotechnology, Beijing Key Laboratory of Cardiometabolic Molecular Medicine, Institute of Molecular Medicine, Peking University, Beijing, 100871 China; 4 Department of Physiology and Biophysics, University of Washington School of Medicine, Seattle, WA 98195 USA

**Keywords:** diabetic beta-cells, calcium signaling alterations, SERCA pump, db/db mice

## Abstract

Upon glucose elevation, pancreatic beta-cells secrete insulin in a Ca^2+^-dependent manner. In diabetic animal models, different aspects of the calcium signaling pathway in beta-cells are altered, but there is no consensus regarding their relative contributions to the development of beta-cell dysfunction. In this study, we compared the increase in cytosolic Ca^2+^ ([Ca^2+^]_i_) via Ca^2+^ influx, Ca^2+^ mobilization from endoplasmic reticulum (ER) calcium stores, and the removal of Ca^2+^ via multiple mechanisms in beta-cells from both diabetic db/db mice and non-diabetic C57BL/6J mice. We refined our previous quantitative model to describe the slow [Ca^2+^]_i_ recovery after depolarization in beta-cells from db/db mice. According to the model, the activity levels of the two subtypes of the sarco-endoplasmic reticulum Ca^2+^-ATPase (SERCA) pump, SERCA2 and SERCA3, were severely down-regulated in diabetic cells to 65% and 0% of the levels in normal cells. This down-regulation may lead to a reduction in the Ca^2+^ concentration in the ER, a compensatory up-regulation of the plasma membrane Na^+^/Ca^2+^ exchanger (NCX) and a reduction in depolarization-evoked Ca^2+^ influx. As a result, the patterns of glucose-stimulated calcium oscillations were significantly different in db/db diabetic beta-cells compared with normal cells. Overall, quantifying the changes in the calcium signaling pathway in db/db diabetic beta-cells will aid in the development of a disease model that could provide insight into the adaptive transformations of beta-cell function during diabetes development.

**Electronic supplementary material:**

The online version of this article (doi:10.1007/s13238-014-0075-7) contains supplementary material, which is available to authorized users.

## INTRODUCTION

Diabetes affects millions of people and exacts a significant toll on both individual health and society as a whole. Because diabetes is associated with both genetic and environmental factors, the etiology of the disease is complicated and unclear. Interestingly, different Ca^2+^ signaling components are disturbed in a wide range of organelles in diabetic animals and patients, suggesting a pivotal role for the dysregulation of Ca^2+^ signaling in the development of diabetes (Bergsten 2000).

Upon blood glucose elevation, pancreatic beta-cells secrete insulin in an intracellular Ca^2+^ concentration ([Ca^2+^]_i_)-dependent manner, which acts on downstream target tissues to facilitate glucose uptake. This process involves different components of the Ca^2+^ signaling pathways. For example, depolarization triggered by glucose metabolism opens L-type voltage-gated Ca^2+^ channels, leading to an initial extracellular Ca^2+^ influx (Rorsman 1997) and the subsequent mobilization of Ca^2+^ stores via pathways such as the IP_3_ and ryanodine receptors (Islam 2002; Duman et al. 2006). Simultaneously, clearance mechanisms such as the sarco/endoplasmic reticulum Ca^2+^-ATPase (SERCA), the plasma membrane Ca^2+^-ATPase (PMCA) and the sodium calcium exchanger (NCX) are activated to reduce elevated [Ca^2+^]_i_ to the physiological resting level (Chen et al. 2003; Hughes et al. 2006). Altered Ca^2+^ signaling is consistently observed in pancreatic beta-cells in diabetic animal models. For example, the L-type calcium channel was up-regulated in both neonatally streptozocin-induced and Goto-Kakizaki (GK) diabetic rats (Kato et al. 1996; Kato et al. 1994) but was down-regulated in islets from other rat models of type II diabetes (Iwashima et al. 2001; Roe et al. 1996). An altered release of endoplasmic reticulum (ER) Ca^2+^ stores via the ryanodine receptor has also been hypothesized to occur during diabetes development (Islam 2002).

SERCA is the major Ca^2+^ extrusion mechanism in mouse and rat pancreatic beta-cells (Chen et al. 2003; Hughes et al. 2006). SERCA pump activity could not be detected in db/db islets (Roe et al. 1994), whereas a selective down-regulation of SERCA3 sub-type mRNA has been reported in GK rats (Varadi et al. 1996). Missense mutations in human SERCA3 have been associated with type II diabetes (Varadi et al. 1999), suggesting a crucial role for SERCA3 in the etiology of diabetes. However, SERCA3-specific knockout (KO) mice are normoglycemic and have normal insulinemia (Arredouani et al. 2002). PMCA activity is down-regulated in certain types of diabetic islets (Roe et al. 1994; Hoenig et al. 1990; Levy et al. 1998), in contrast to beta-cells cultured under high glucose conditions, which exhibited enhanced NCX transcription (Ximenes et al. 2003). These controversies can only be resolved if the functions of different Ca^2+^ signaling proteins in normal and diabetic beta-cells are systematically compared under identical experimental conditions.

To address these questions, we compared calcium clearance in age-matched pancreatic beta-cells that were isolated from both C57BL/6J control mice and db/db mice, a widely used type II diabetic mouse model. The changes can be quantitatively modeled as a 35% reduction in SERCA2 activity, a full inhibition of the SERCA3 pump, a 30% increase in the NCX capacity and a 27% reduction in Ca^2+^ influx. Despite the severely compromised SERCA function, the Ca^2+^ concentration in the ER ([Ca^2+^]_ER_) was reduced only slightly, to 89% of the control, while the ER mobilization pathways remained unchanged. Overall, these changes led to significant alterations in the glucose-induced calcium oscillations in the beta-cells of the db/db mice relative to the control.

## RESULTS

### Ca^2+^ clearance after depolarization stimulated Ca^2+^ influx in normal and db/db beta-cells

**Figure 1:**
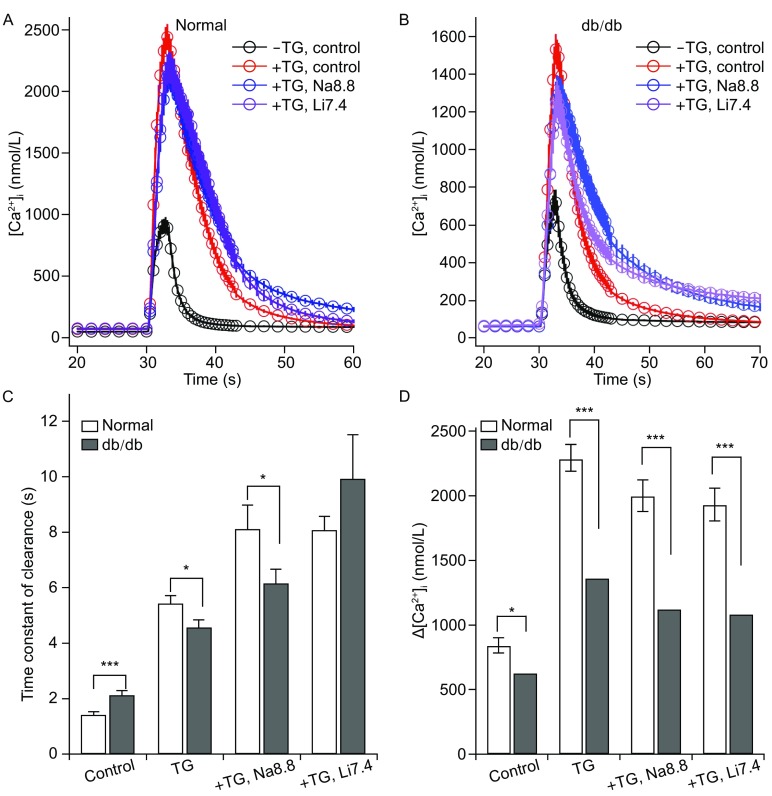
**Ca** ^**2+**^ **recoveries after short depolarizations in normal and db/db diabetic beta-cells**. (A) Averaged clearances in normal beta-cells pretreated with 1 μmol/L TG in control solution (*n* = 42, red), Li7.4 solution (*n* = 21, blue) and Na8.8 solution (*n* = 22, purple) and in cells that were not pretreated with TG (*n* = 25, dark). (B) Averaged clearances in db/db beta-cells pretreated with 1 μmol/L TG in control solution (*n* = 32, red), Li7.4 solution (*n* = 16, blue) and Na8.8 solution (*n* = 16, purple) and in cells that were not pretreated with TG (*n* = 34, dark). (C) Summary of the recovery time constants in different solutions from normal and db/db beta-cells. Individual calcium recovery traces were fitted with single-exponential functions. (D) Summary of KCl-triggered [Ca^2+^]_i_ elevations ([Ca^2+^]_i_) in different solutions from normal and db/db beta-cells

We have previously dissected the contributions of multiple clearance mechanisms in pancreatic beta-cells isolated from Balb/c mice through pharmacological manipulation (Chen et al. 2003). In this work, we used this method to compare the clearance mechanisms in beta-cells isolated from age-matched db/db and C57BL/6J mice. To quantitatively evaluate Ca^2+^ clearance after depolarization, control and db/db cells were stimulated with 70 mmol/L KCl for 3 s and then switched to normal extracellular solution (Fig. 1A and 1B). Compared with normal beta-cells, the db/db cells exhibited a reduced depolarization-triggered [Ca^2+^]_i_ elevation, and [Ca^2+^]_i_ returned to the basal level in normal extracellular solution at a significantly slower rate. In cells that had been pre-treated with thapsigargin (TG), an irreversible inhibitor of the SERCA pump, the increase in [Ca^2+^]_i_ remained smaller, but the clearance was faster in db/db cells than it was in the control cells (Fig. 1C and 1D). This result suggested not only an impairment of the SERCA pump but also a possible up-regulation of clearance mechanisms other than the SERCA pump in db/db beta-cells. To dissect the contributions of NCX and PMCA in clearance, we switched to a Na^+^-free solution (Li7.4) or a high pH extracellular solution (Na8.8) after depolarization in cells pretreated with TG (Chen et al. 2003). Compared with the control cells, Ca^2+^ clearance in db/db cells was much faster in the presence of SERCA and PMCA inhibition (TG + Na8.8). In contrast, [Ca^2+^]_i_ returned to the basal level with the same kinetics in db/db and control cells when only the PMCA pump was functioning (TG + Li7.4). Taken together, these results indicate the selective down-regulation of depolarization-induced Ca^2+^ influx and the SERCA pump and the up-regulation of NCX in db/db cells.

### Reduced function of the SERCA pump in db/db beta-cells compared with the control

**Figure 2:**
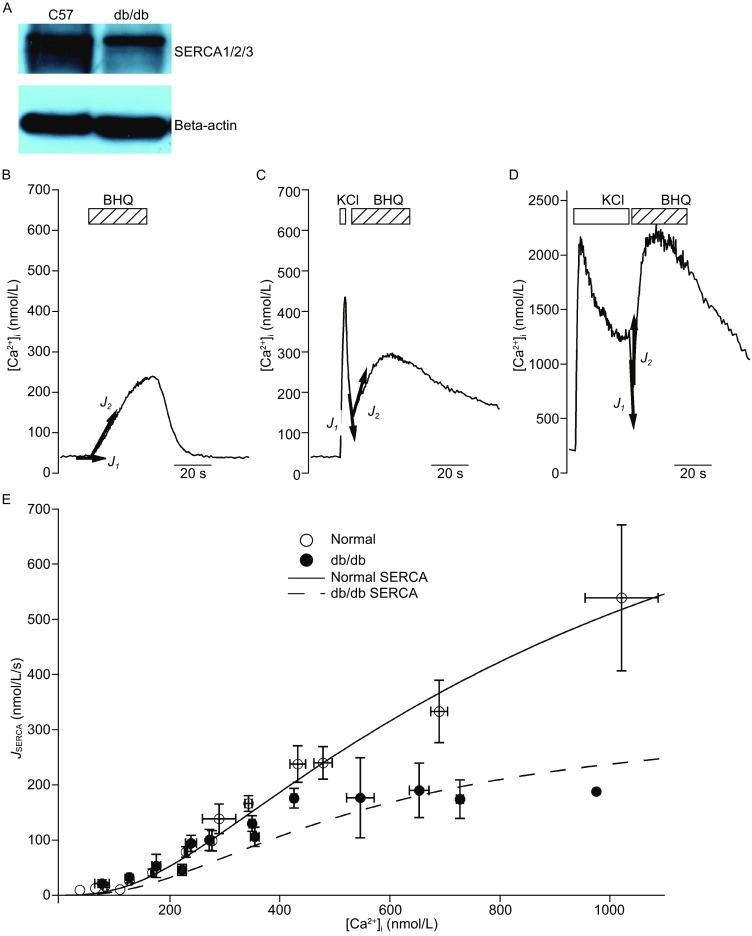
**Impaired SERCA function in beta-cells from db/db mice**. (A) Reduced total SERCA protein levels in islets from db/db mice compared to those from normal mice. Beta-actin was used as an internal control, and the figure is representative of three independent repeats. (B–D) BHQ (100 μmol/L) was acutely applied to resting cells (B) and cells that had been previously stimulated with KCl for 3 s (C) or 30 s (D). The total cellular Ca^2+^ flux (defined as –d[Ca^2+^]_i_/dt) before (*J*_*1*_) and after (*J*_*2*_) BHQ application was plotted on each figure. (E) *J*_*SERCA*_ from normal beta-cells (open circle, *n* = 256) and from db/db diabetic beta-cells (filled circle, *n* = 119) as a function of [Ca^2+^]_i_. The line indicates the total SERCA activity in normal beta-cells (including both SERCA2 and SERCA3 activity) according to the model, whereas the dashed line represents the activity in db/db cells

The relative expression levels of the SERCA pump in normal and db/db islets were studied using a SERCA-specific antibody that recognizes all three isoforms of SERCA. In agreement with previous experiments (Roe et al. 1994; Varadi et al. 1996), the SERCA protein levels were severely reduced in db/db islets compared with normal islets (Fig. 2A). Because the Western blotting experiments were performed using whole islets that contain alpha- and beta-cells, it is difficult to determine the specific reduction in SERCA in beta-cells. Therefore, we took a direct approach to measure SERCA function in live beta-cells (Duman et al. 2006; Albrecht et al. 2002). The application of a high concentration of a rapid inhibitor of the SERCA pump, BHQ (100 μmol/L), in resting cells (Fig. 2B) or in cells that were stimulated with a short (3 s, Fig. 2C) or long (30 s, Fig. 2D) depolarization, produced an upstroke in the [Ca^2+^]_i_ trace. The difference between the BHQ-induced rate of change in [Ca^2+^]_i_ (J_2_) and the initial slope of the [Ca^2+^]_i_ trace immediately before BHQ application (J_1_) represented the SERCA-dependent uptake of cytosolic Ca^2+^, which was correlated with different [Ca^2+^]_i_ levels (Duman et al. 2006; Albrecht et al. 2002). SERCA activity was inhibited at [Ca^2+^]_i_ ranging from 200 nm to 1000 nm, with increased inhibition at higher [Ca^2+^]_i_ (Fig. 2D).

### Quantitative modeling of the alterations in Ca^2+^ influx and clearance in db/db beta-cells

**Figure 3:**
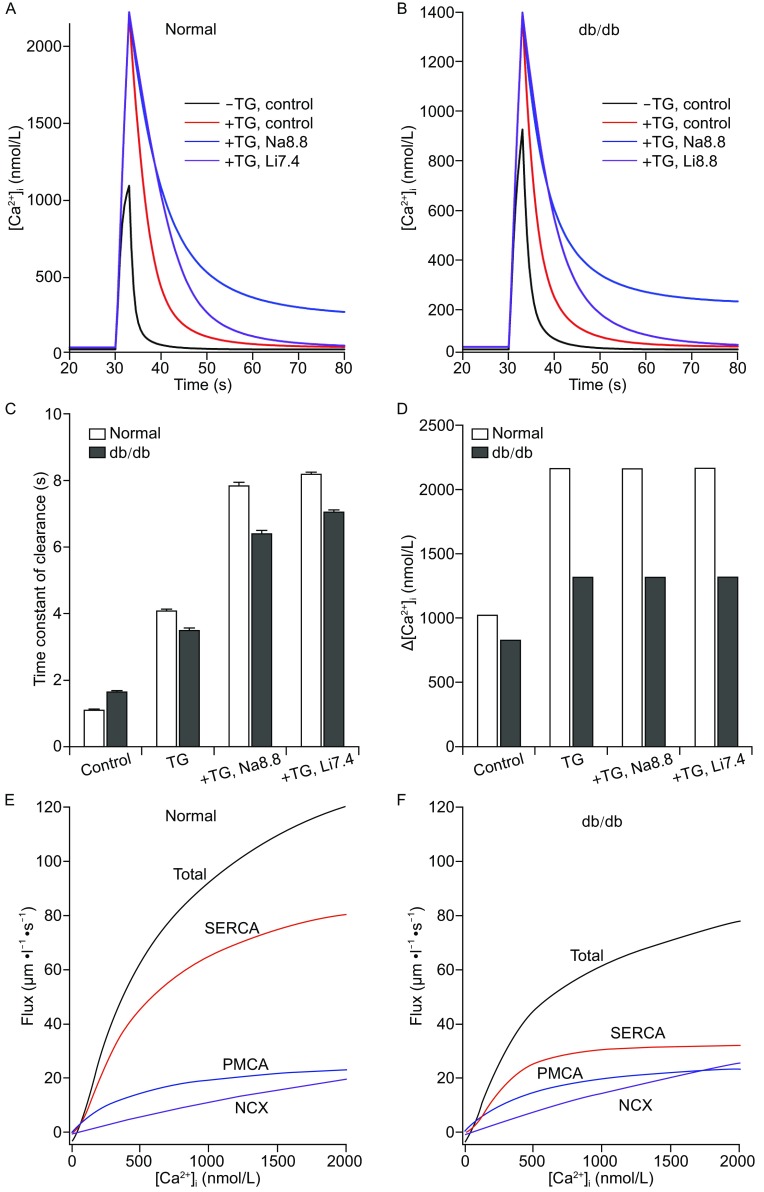
**Simulated Ca** ^**2+**^ **recoveries after short depolarizations in normal and db/db diabetic beta-cells**. (A and B) Simulated clearances under different conditions obtained from normal beta-cells (A) and db/db diabetic beta-cells (B). (C and D) Simulated recovery time constants (C) and KCl-triggered [Ca^2+^]_i_ elevations (D) in beta-cells from normal and db/db mice. (E and F) Simulated kinetic model for Ca^2+^ transport in normal (E) and db/db (F) beta-cells. The flux rates were much higher than the rates of [Ca^2+^]_i_ change shown in Fig. 2E because the model includes the effects of strong Ca^2+^ binding in the cytoplasm by endogenous buffers and fluorescent indicators

Previously, we built a mathematical model to simulate calcium clearance in normal beta-cells that used the K_d_ for SERCA2 for SERCA in the simulation (Chen et al. 2003). However, pancreatic beta-cells also express the SERCA3 subtype (Varadi et al. 1996), which has a much lower affinity for Ca^2+^ (1100 nmol/L) than does SERCA2 (270 nmol/L) (Lytton et al. 1992). We therefore revised our model to accommodate two SERCA subtypes and manipulated the maximal flux rates through the influx and clearance mechanisms to best simulate the peaks of transient [Ca^2+^]_i_ and the time constants following depolarization in normal cells under different conditions (Fig. 3A). This simulation yielded a Vmax_SERCA2_:Vmax_SERCA3_ ratio of approximately 1.2:1, and the total SERCA activity correlated well with the experimental data throughout a wide range of [Ca^2+^]_i_ (Fig. 2D, dark dashed lines). By comparing the experimental depolarization-evoked peak [Ca^2+^]_i_ elevations in the control and db/db beta-cells pretreated with TG, we determined that a 27% decrease in Ca^2+^ influx from db/db cells was required to account for the decrease in amplitude triggered by depolarization. Based on the clearance dynamics in normal and db/db cells treated with TG + Li7.4 and TG + Na8.8, we determined that the NCX activity in db/db cells was up-regulated 130% relative to control cells. The residual activity levels of SERCA2 and SERCA3 in db/db beta-cells were 65% and 0% of their respective levels in the control cells, which approximated the experimental data nicely (Fig. 2D, red dashed line). With these altered parameters, the simulated [Ca^2+^]_i_ elevation and the clearance dynamics after depolarization fit the experimental data relatively well (Fig. 3B–D). With the parameters chosen, we reproduced the relative flux rates of the model for normal and beta-cells from db/db mice (Fig. 3E and 3F). The total Ca^2+^ extrusion rate in db/db beta-cells was ~66% of the control, and the relative role of SERCA was also reduced (from ~66%–71% of the total flux to ~41%–56%). In contrast, the relative contribution of the NCX increased, especially at high [Ca^2+^]_i_ (>900 nm), indicating that NCX hyper-activity may compensate for the loss of the SERCA3 subtype.

### No alterations in ER Ca^2+^ permeability in db/db beta-cells compared to the control

**Figure 4:**
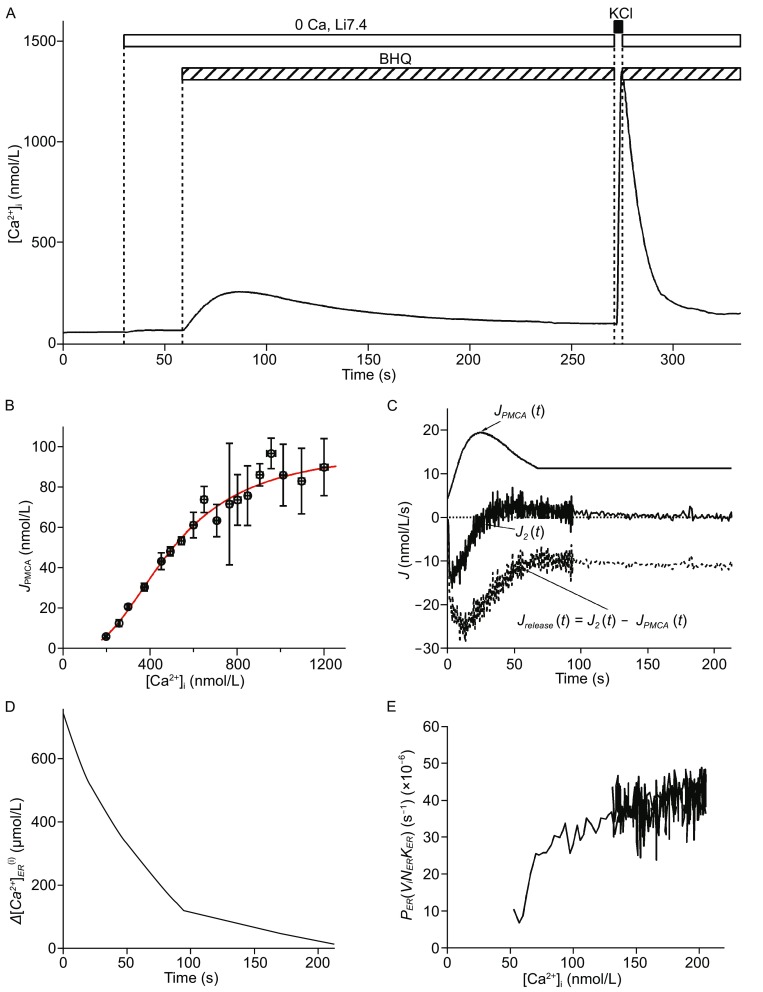
**Detection of Ca** ^**2+**^ **content in the ER and ER Ca** ^**2+**^ **permeability in single beta-cells**. (A) A cell was initially bathed in Ca^2+^-free and Na^+^-free solution, then switched to a solution containing 100 μmol/L BHQ to block the SERCA pump. After depletion of the Ca^2+^ in the ER, KCl (70 mmol/L) with Ca^2+^ (4 mmol/L) was applied to the cell for 3 s to induce a transient increase in [Ca^2+^]_i_. The clearance dynamics thereafter were mainly due to the removal of Ca^2+^ by the PMCA (*J*_*PMCA*_). (B) The correlation of *J*_*PMCA*_ with [Ca^2+^]_i_ obtained from (A) can be fitted with a Hill function. (C) Time course of J_PMCA_, total cytoplasmic Ca^2+^ flux (*J*_*2*_) and *J*_*release*_ after BHQ application. *J*_*release*_ was calculated as the difference between the *J*_*2*_ and the simulated *J*_*PMCA*_ at different time points after BHQ application. (D) Plot of time course of ${\Delta} \left[ {Ca^{2+ } } \right]_{ER}^{(t)} (t)$ after BHQ application, which was calculated as stated in the main text (Albrecht et al. 2002). (E) Relationship of *P*_*ER*_ *[v*_*i*_ */(v*_*ER*_ *k*_*ER*_ *)]* to different [Ca^2+^]_i_ obtained during the BHQ-stimulated ER calcium release

SERCA pump defects combined with an up-regulation of NCX function often result in reduced uptake of Ca^2+^ to ER calcium stores (Ximenes et al. 2003), and alterations in ER permeability during diabetes have been suggested (Islam 2002). To quantitatively evaluate the Ca^2+^ content in the ER and its permeability, we modified a protocol from a previous study conducted in sympathetic neurons (Albrecht et al. 2002). Cells were initially bathed in an extracellular solution that did not contain Ca^2+^ or Na^+^ (Li7.4) to block the NCX and Ca^2+^ influx and were then perfused with BHQ to trigger Ca^2+^ release from the ER calcium stores (Fig. 4A). After [Ca^2+^]_i_ returned to the basal level, the cells were depolarized for 3 s in the presence of 4 mmol/L Ca^2+^ to boost the Ca^2+^ influx. Finally, the extracellular solution was changed back to the Ca^2+^- and Na^+^-free solution with BHQ. The rate of Ca^2+^ extrusion after KCl depolarization was therefore due solely to the PMCA (*J*_*PMCA*_) and could be described by a Hill function that correlated with the different [Ca^2+^]_i_ levels (Fig. 4B). Because the calcium-flux changes induced by the first BHQ application (*J*_*BHQ*_(t)) were a result of both the release from the ER (*J*_*release*_) and Ca^2+^ extrusion via the PMCA, the *J*_*release*_(t) was calculated as the difference between *J*_*BHQ*_ and *J*_*PMCA*_ at each time point (Fig. 4C). The drop in the ER Ca^2+^ concentration ([Ca^2+^]_ER_) at different time points was calculated based on the following equation:

$$ {\Delta} \left[ {Ca^{2+ } } \right]_{ER} \left( t \right) = - \frac{{v_{i} }}{{v_{ER}\, \kappa_{ER} }}\int\limits_{t}^{end} {J_{release}\, \kappa_{i} dt} = \frac{{v_{i} }}{{v_{ER}\, \kappa_{ER} }}{\Delta} \left[ {Ca^{2+ } } \right]_{ER}^{(i)} \left( t \right), $$

(Albrecht et al. 2002), in which *v*_*i*_ and *v*_*ER*_ are the volumes of the cytoplasm and the ER, respectively, and κ_i_ and κ_ER_ are the calcium buffering ratios of the cytoplasm and the ER, respectively. The minus of the integral (${\Delta} \left[ {Ca^{2+ } } \right]_{ER}^{\left( i \right)} \left( t \right)$) was the change in total cytoplasmic Ca^2+^ concentration that would occur if from time *t* onward, *J*_*release*_ were deposited into a closed compartment that has the same volume as the cytoplasm. Assuming that [Ca^2+^]_ER_ equilibrates with [Ca^2+^]_i_ after BHQ application, we calculated the initial content in the ER according to the following equation:

$$ {\Delta} \left[ {Ca^{2+ } } \right]_{ER}^{i} \left( 0 \right) = \left[ {Ca^{2+ } } \right]_{i,end} \kappa_{i} + \int\limits_{0}^{end} {J_{release}\, \kappa_{i} dt} $$

Therefore, the time course of ${\Delta} \left[ {Ca^{2+ } } \right]_{ER}^{\left( i \right)} \left( t \right)$ was shown in Fig. 4D, and the relative permeability of the ER was estimated according to the equation

$P_{ER} \left( t \right)\left[ {\frac{{v_{i} }}{{v_{ER} \kappa_{ER} }}} \right] \approx - \frac{{J_{release} \left( t \right)}}{{{\Delta} \left[ {Ca^{2+ } } \right]_{ER}^{\left( i \right)} \left( t \right)}}$ (Fig. 4E) (Albrecht et al. 2002).

**Figure 5:**
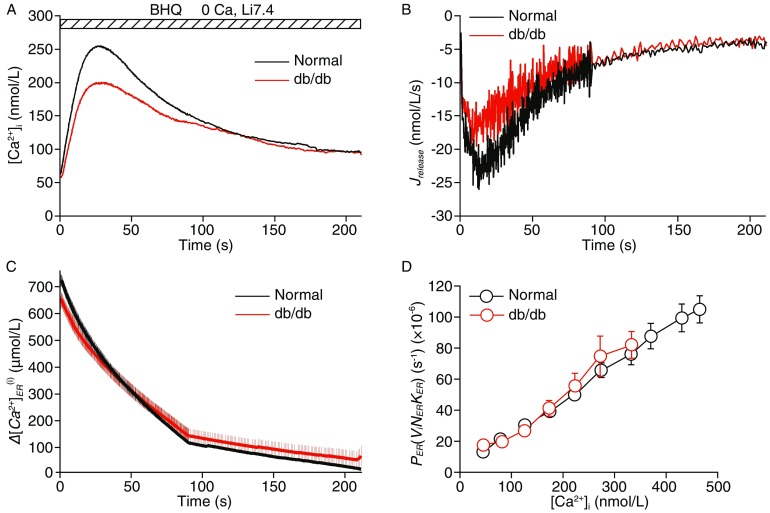
**Reduced** ${\Delta} \left[ {Ca^{2+ } } \right]_{ER}^{(i)}$ **and unaltered ER Ca** ^**2+**^ **permeability in db/db beta-cells**. (A) Average BHQ-triggered [Ca^2+^]_i_ transients in Ca^2+^-free and Na^+^-free solutions from normal beta-cells (*n* = 43, dark) and db/db diabetic beta-cells (*n* = 31, red). (B) Time courses of average *J*_*release*_ in normal and db/db cells. (C) Reduced initial ${\Delta} \left[ {Ca^{2+ } } \right]_{ER}^{(i)}$ in db/db diabetic beta-cells relative to normal cells (*P* < 0.05). (D) The average relative ER permeability during the BHQ stimulation was approximately the same in normal and db/db diabetic beta-cells

In beta-cells from db/db mice, the BHQ-triggered [Ca^2+^]_i_ transient was significantly lower than in the control cells (Fig. 5A). Based on the calculations, the average $\left[ {Ca^{2+ } } \right]_{ER}^{i}$ in the db/db beta-cells was approximately 89% of the value in the normal cells (Fig. 5C, *P* < 0.05). Although the time courses of *J*_*release*_ were apparently different in different cell types (Fig. 5B), the relative permeabilities of the ER were approximately the same (Fig. 5D), suggesting that the difference in ER release was due to a difference in the initial [Ca^2+^]_ER_.

### Glucose-induced calcium oscillations in normal and db/db beta-cells

**Figure 6:**
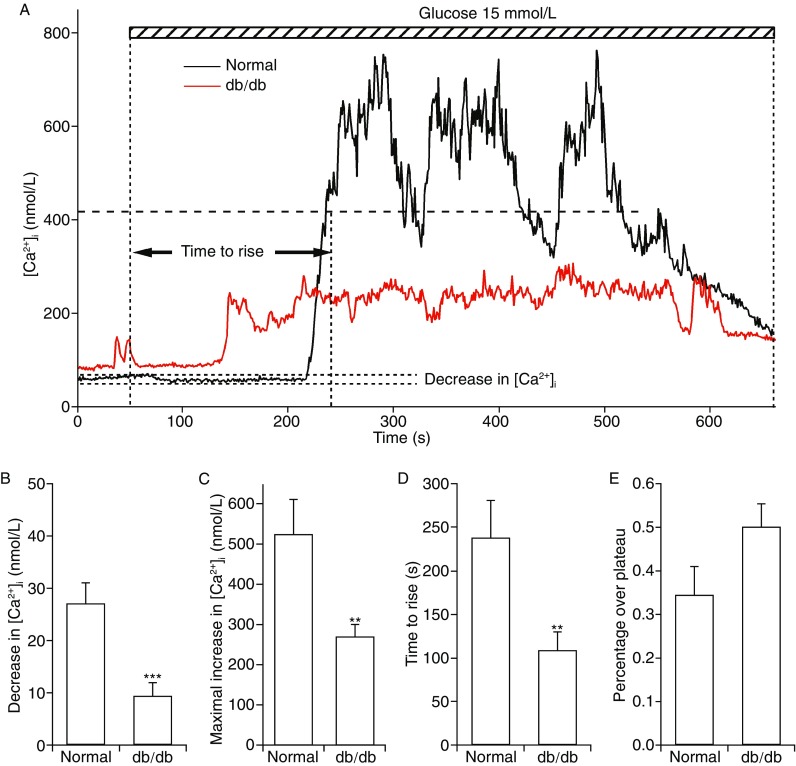
**Altered glucose-triggered calcium oscillations in db/db beta-cells**. (A) Representative examples of calcium oscillations triggered by glucose (15 mmol/L) in one beta-cell from normal mice (dark) and in one beta-cell from db/db mice (red). The initial decrease in [Ca^2+^]_i_ was quantified as the difference between the resting [Ca^2+^]_i_ and the minimal [Ca^2+^]_i_ reached within 120 s after glucose perfusion. The maximal increase in [Ca^2+^]_i_ was calculated as the difference between the resting [Ca^2+^]_i_ and the maximal [Ca^2+^]_i_ reached within the period of glucose perfusion. The time to rise was defined as the time delay between the perfusion of glucose and the time when [Ca^2+^]_i_ reached 50% of the maximal increase in [Ca^2+^]_i_. Finally, the percentage over plateau of each trace was defined as the time during which the [Ca^2+^]_i_ was greater than 50% of the maximal increase divided by the total time of glucose stimulation. (B, C, D, E) Compared to normal cells (*n* = 23), pancreatic beta-cells from db/db mice (*n* = 21) exhibited a reduction in the average initial decrease in [Ca^2+^]_i_ (B), a reduction in the average maximal increase in [Ca^2+^]_i_ (C), and an accelerated response to glucose (D). Although db/db cells spent a slightly greater percentage of time at [Ca^2+^]_i_ greater than 50% of the maximal increase in [Ca^2+^]_i_ during glucose stimulation, the average difference compared to normal cells was not significant (E)

Finally, we directly monitored the calcium oscillations induced by 15 mmol/L glucose in normal and db/db beta-cells (Fig. 6A). Upon the application of glucose, [Ca^2+^]_i_ often decreased slightly before being elevated, which was suggested to be due to the ATP-activated SERCA-dependent sequestration of [Ca^2+^]_i_ in the ER (Marie et al. 2001). Consistent with a significant inhibition of SERCA2 activity (Fig. 3), the initial decrease in [Ca^2+^]_i_ was reduced significantly in db/db cells (Fig. 6B). The maximal elevation in [Ca^2+^]_i_ triggered by glucose was also reduced in db/db cells compared with the control cells. However, the glucose-stimulated [Ca^2+^]_i_ increase was accelerated in the db/db cells, and the percentage of time the cells spent over the [Ca^2+^]_i_ plateau was slightly, but not significantly, different in the db/db cells. Overall, our data suggest that multiple characteristics of the glucose-triggered calcium transient are altered in db/db beta-cells.

## DISCUSSION

In the current study, we showed that the PMCA activity in islets from db/db mice was not significantly different compared with that observed in normal mice, in contrast to the previously reported down-regulation of PMCA function in db/db islets and islets with insulin resistance (Roe et al. 1994; Hoenig et al. 1990; Levy et al. 1998; Alzugaray et al. 2009). The difference may be due to the different experimental conditions tested and the different cell models used. However, because PMCA plays a minor role in calcium clearance after depolarization in both mouse and rat pancreatic beta-cells (Chen et al. 2003; Hughes et al. 2006), we believe that PMCA may not be the primary target in the reshaping of beta-cell calcium signaling pathways during the development of diabetes. The functions of the SERCA2 and SERCA3 subtypes were both impaired in db/db beta-cells, as opposed to the selective impairment of SERCA3 function in GK rats. This result partially explains the normoglycemic phenotype of SERCA3 KO mice (Arredouani et al. 2002). Because the high-affinity SERCA2 subtype functions in basal [Ca^2+^]_i_ regulation in beta-cells (Arredouani et al. 2002), its down-regulation correlates with the reduction in the glucose-stimulated initial decrease in [Ca^2+^]_i_ in db/db beta-cells (Fig. 6B), which may perturb glucose-stimulated insulin secretion (Roe et al. 1994; Marie et al. 2001). In addition, we report for the first time a 30% increase in NCX activity in db/db beta-cells, which is consistent with the enhancement of NCX transcription observed in islets cultured in high glucose (Ximenes et al. 2003). Thus our data generally agree with previous studies conducted in this field. In addition, by fitting the experimental data to a modified mathematical model, we obtained a quantitative description of all alterations in the calcium signaling pathways in db/db mice, which has not been previously reported.

Perturbed Ca^2+^ homeostasis has long been proposed as a hallmark of diabetes (Bergsten 2000). Despite the many alterations in the calcium signaling pathway in beta-cells from db/db mice, we propose that the down-regulation of SERCA is the earliest event. SERCA2 and SERCA3 expression levels are reduced in islets from both type I and type II diabetic mice at earlier stages (Varadi et al. 1996; Liang et al. 2011), which may be due to increased levels of glucose (Levy et al. 1998), saturated fatty acids (Cunha et al. 2008), and cytokines (Dula et al. 2010; Cardozo et al. 2005) in the blood vessels and enhanced insulin resistance in beta-cells (Borge et al. 2002). Because the SERCA pump is the dominant clearance mechanism in beta-cells that limits the amplitude of [Ca^2+^]_i_ transients after depolarization, SERCA pump inhibition dramatically enhances depolarization-induced insulin secretion (Chen et al. 2003; Hughes et al. 2006). Therefore, the down-regulation of SERCA is a beneficial adaptation mechanism that enables beta-cells to secrete more insulin to compensate for the loss of beta-cell mass in type I diabetes or to overcome insulin resistance in type II diabetes. This explanation is supported by the accelerated onset of the glucose-stimulated Ca^2+^ transient in db/db diabetic beta-cells relative to the control cells (Fig. 6B). Along with the reduction in SERCA expression, fine-tuning of the voltage-gated calcium channel currents is needed to generate suitable [Ca^2+^]_i_ transients that maximize the secretion response and minimize the apoptosis triggered by high [Ca^2+^]_i_. Because the NCX contributes more to the total clearance mechanism of beta-cells at relatively high [Ca^2+^]_i_ (Chen et al. 2003), the loss of low-affinity SERCA3 may lead to the adaptive up-regulation of NCX observed here, which assists in calcium clearance from the cytosol during the stimulation and shaping of glucose-triggered calcium transients. Despite the significant reduction in SERCA activity and the enhanced NCX activity, the decrease in [Ca^2+^]_ER_ (11%) in db/db beta-cells was relatively small, in contrast to the severe reduction in [Ca^2+^]_ER_ from insulin-secreting cell lines treated acutely or chronically with saturated fatty acids (Cunha et al. 2008; Gwiazda et al. 2009). These results highlight the importance of systematic investigations of calcium signaling pathways alterations in primary beta-cells isolated from diabetic animals, rather than solely detecting alterations in beta-cells cultured in conditions that mimic diabetes. Decreased [Ca^2+^]_ER_ is a susceptibility factor for ER stress (Cunha et al. 2008; Cardozo et al. 2005). Therefore, in the long run, a prolonged small decrease in [Ca^2+^]_ER_ in combination with other ER stress susceptibility factors, such as excessive insulin biosynthesis (Grill and Bjorklund 2001), may ultimately result in beta-cell failure, apoptosis, and severe glucose intolerance.

In conclusion, we characterize changes in multiple aspects of the calcium signaling pathway in beta-cells isolated from db/db mice compared with the control. These quantitative studies will help build better models to describe glucose-induced Ca^2+^ oscillations in diabetic beta-cells (Bertram et al. 2004), which will provide insights into the development of insulin secretion dysfunction and beta-cell failure in the development of diabetes.

## MATERIALS AND METHODS

### Cell culture and Western blotting experiments

Primary islets and beta-cells were isolated from age-matched C57BL/6J and db/db mice (7–8 weeks) as previously described (Chen et al. 2003). Mouse handling and experimental procedures were conducted in accordance with the Committee for the Use of Live Animals in Teaching and Research at Institute of Molecular Medicine, Peking University. The protocol was approved by the Committee on the Ethics of Animal Experiments of Peking University (Permit Number: IMM-ChenLY-1). All surgery was performed under chloral hydrate anesthesia, and all efforts were made to minimize suffering. At this age, db/db mice were overweight and glucose intolerant, as previously reported (Kobayashi et al. 2000). The isolated cells were plated onto polyornithine-coated glass coverslips and cultured at 37°C in 5% CO_2_ in RPMI 1640 culture medium containing 10 mmol/L glucose, 10% fetal bovine serum, 100 µg/mL streptomycin and 100 IU/mL penicillin. Pancreatic beta-cells were identified by size (Cho et al. 2010) and generally used on culture day 2 or 3. For the Western blotting experiments (Fig. 2), isolated islets were homogenized using homogenate buffer to obtain total proteins. Equal amounts of proteins (approximately 30 µg/lane) were loaded to SDS-PAGE and analyzed by Western blotting using anti-SERCA1/2/3 (SANTA CRUZ, 1:800) and anti-beta actin antibody (Sigma, St. Louis, MO, 1:2000). The incubation with the antibodies was followed by the application of rabbit anti-mouse IgG peroxidase conjugate (Sigma, St. Louis, MO, 1:5000) and goat anti-rabbit IgG (Perkin Elmer, 1:5000). The blots were then probed with Western Lighting plus-ECL (Perkin Elmer, Inc.) (He et al. 2008).

### Experimental set-up and Ca^2+^ photometry

Cells were loaded with fura-2-AM (10 µmol/L) in modified Ringer’s solution (130 mmol/L NaCl, 2.5 mmol/L KCl, 1 mmol/L MgCl_2_, 2 mmol/L CaCl_2_, 4 mmol/L glucose, and 10 mmol/L HEPES, pH 7.3) at room temperature for 20–25 min. The modified Ringer’s solution was also used as the extracellular solution for basal recording in the glucose-induced calcium oscillation experiments, in which 15 mmol/L glucose replaced 4 mmol/L glucose as the stimulus. For the Ca^2+^ clearance/[Ca^2+^]_ER_ evaluation experiment, the cells were perfused with the modified Ringer’s solution containing 15 mmol/L (instead of 4 mmol/L) glucose and 250 µmol/L diazoxide, and various reagents were added to this solution to inhibit clearance mechanisms, as described in the individual experiments. The depolarization solution (KCl) consisted of 70 mmol/L KCl, 67 mmol/L NaCl, 4 mmol/L CaCl_2_, 1 mmol/L MgCl_2_, 15 mmol/L glucose, 250 µmol/L diazoxide, and 10 mmol/L HEPES, pH 7.3. To inhibit the NCX, we used a Na^+^-free solution in which Li^+^ replaced Na^+^ (Li7.4) and raised the pH of the solution to 8.8 (Na8.8) to slow the PMCA pump (Chen et al. 2003). Rapid solution changes (<500 ms) were digitally controlled by a fast local perfusion system (Chen et al. 2003).

For photometry, the cells were excited by sequential 340 and 380 nm light generated by a computer-controlled PolyChrome IV (TILL Photonics) light source, and the emission at 505 nm was collected by a photodiode. The PULSE software was used to coordinate protocols and collect data, which were analyzed using IGOR Pro. The standard calibration parameters R_min_, R_max_ and K* were determined as previously described (Chen et al. 2003).

### Measurement of J_SERCA_ and J_release_ activity at different [Ca^2+^]_i_ in live beta-cells

Following previously published reports (Duman et al. 2006; Albrecht et al. 2002), we applied a high dose of tert-butylhydroquinone (BHQ, 100 μmol/L) to abruptly stop the uptake of Ca^2+^ by the ER via the SERCA pump. The total cellular Ca^2+^ flux can be calculated as the rate of change of [Ca^2+^]_i_ (defined as –d[Ca^2+^]_i_/dt). Prior to the application of BHQ, the total cellular Ca^2+^ flux (*J*_*1*_) can be described by the equation

Eq. 1 $$ J_{1} = J_{SERCA} + J_{release} + J_{PM} $$

*J*_*SERCA*_ is the Ca^2+^ flux due to the BHQ-sensitive pumping of Ca^2+^ into the ER; *J*_*release*_ is the flux into the cytosol from intracellular stores; and *J*_*PM*_ is the flux across the plasma membrane. The acute application of BHQ changes the new total cellular Ca^2+^ flux (*J*_*1*_) according to

Eq. 2 $$ J_{2} = J_{release} + J_{PM} $$

Therefore, the value of *J*_*SERCA*_ was calculated as the difference between *J*_*1*_ and *J*_*2*_. In Fig. 4, we briefly treated the beta-cells with High K^+^ solution containing BHQ to obtain Ca^2+^ clearance by the PMCA pump alone, and *J*_*PMCA*_ was fitted to a Hill equation to describe its relationship to different [Ca^2+^]_i_ levels. Therefore, a different *J*_*releas*e_(*t*) was calculated based on Eq. 2, and the appropriate value of *J*_*PMCA*_ was inserted.

### Data analysis

All data were analyzed using the Igor Pro software (Wavemetrics, Lake Oswego, OR). The averaged results are presented as the mean value ± SEM of the number of experiments indicated. The statistical significance was evaluated using either Student’s *t*-test for single Gaussian distributed datasets or the Mann-Whitney rank sum test for non-single Gaussian-distributed datasets. The asterisks *, **, and *** denote statistical significances with *P* values less than 0.05, 0.01, and 0.001, respectively.

## Electronic supplementary material

Below is the link to the electronic supplementary material.

Supplementary material 1 (PDF 30 kb)
