# Supplementary material for: Alterations of the Ca2+ signaling pathway in pancreatic beta-cells isolated from db/db mice
Source: Protein Cell. 2014 Jul 24;5(10):783–94. doi: 10.1007/s13238-014-0075-7 (PMC4180459; doi:10.1007/s13238-014-0075-7)
Supplement: Supplementary file 1 — Supplementary material 1 (PDF 30 kb) [file 13238_2014_75_MOESM1_ESM.pdf]

ONLINE SUPPLEMENTARY MATERIAL

Calculations to estimate the total amount of Ca^{2+} released during the ER Ca^{2+} content and the ER Ca^{2+} permeability.

Cells were bathed in a series of solution changes to evoke two kinds of $[Ca^{2+}]_i$ transients as described in text and Fig. 4A. The first represented Ca^{2+} released from the ER when SERCA was blocked with BHQ and the second following a KCl depolarization permitted calculation of extrusion by the PMCA (J_{PMCA}) as a function of $[Ca^{2+}]_i$ (Fig. 4B). Because the calcium flux changes induced by the initial BHQ application ($J_{BHQ}(t)$) were a result of both release from the ER ($J_{release}$) and Ca^{2+} extrusion via the PMCA, the $J_{release}(t)$ had to be calculated as the difference between J_{BHQ} and J_{PMCA} at each time point (Fig. 4C). The drop in the ER Ca^{2+} concentration ($[Ca^{2+}]_{ER}$) at different time points was calculated based on the equation:

$$\Delta\left[Ca^{2+}\right]_{ER}(t) = -\frac{v_i}{v_{ER}\kappa_{ER}}\int_t^{end} J_{release}\kappa_i dt = \frac{v_i}{v_{ER}\kappa_{ER}}\Delta\left[Ca^{2+}\right]_{ER}^i(t)$$

(21), in which v_i and v_{ER} are the volumes of the cytoplasm and the ER, respectively, and κ_i and κ_{ER} are the calcium binding ratios of the cytoplasm and the ER, respectively. Assuming that $[Ca^{2+}]_{ER}$ equilibrates with $[Ca^{2+}]_i$ after BHQ application, we calculated the initial content in the ER according to the equation:

$$\left[Ca^{2+}\right]_{ER}^i(0) = \left[Ca^{2+}\right]_{i,end}\kappa_i + \int_0^{end} J_{release}\kappa_i dt$$

The change in $[Ca^{2+}]_{ER}$ over time is defined as

$$\left[Ca^{2+}\right]_{ER}^i(t) = \left[Ca^{2+}\right]_{ER}^i(0) - \int_0^{end} J_{release}\kappa_i dt\ ,$$

as shown in Fig. 4D. The relative permeability of the ER was estimated according to the equation,

$$P_{ER}(t)\left[\frac{v_i}{v_{ER}\kappa_{ER}}\right] \approx -\frac{J_{release}(t)}{\left[Ca^{2+}\right]_{ER}^i(t)}$$ (Fig. 4E) (21).
